# 1-(4-Chloro­benzo­yl)-3-cyclo­hexyl-3-methyl­thio­urea

**DOI:** 10.1107/S1600536811025013

**Published:** 2011-07-02

**Authors:** Aisha A. Al-abbasi, Bohari M. Yamin, Mohammad B. Kassim

**Affiliations:** a School of Chemical Sciences & Food Technology, Faculty of Science & Technology, Universiti Kebangsaan Malaysia, 43600 Selangor, Malaysia; b Fuel Cell Institute, Universiti Kebangsaan Malaysia, 43600 Selangor, Malaysia

## Abstract

In the title compound, C_15_H_19_ClN_2_OS, the dihedral angle between the amide and thio­urea fragments is 58.07 (17)°. The cyclo­hexane group adopts a chair conformation and is twisted relative to the thio­urea fragment, forming a dihedral angle of 87.32 (18)°. In the crystal, N—H⋯S hydrogen bond links the mol­ecules into chains running parallel to the *a*-axis direction.

## Related literature

For related structures and background references, see: Al-abbasi & Kassim (2011 ▶); Nasir *et al.* (2011 ▶). For further synthetic details, see: Hassan *et al.* (2008 ▶).
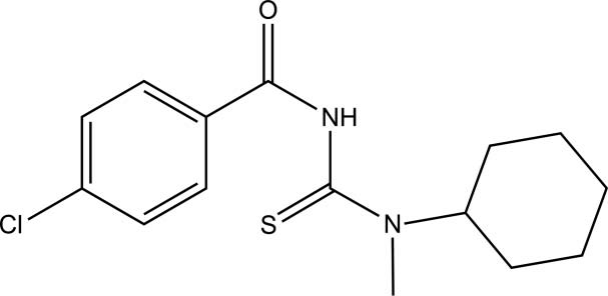


## Experimental

### 

#### Crystal data

C_15_H_19_ClN_2_OS
*M* *_r_* = 310.83
Triclinic, 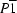

*a* = 5.042 (2) Å
*b* = 11.368 (4) Å
*c* = 15.139 (6) Å
α = 69.865 (7)°
β = 82.698 (8)°
γ = 80.702 (8)°
*V* = 801.7 (5) Å^3^
*Z* = 2
Mo *K*α radiation
μ = 0.37 mm^−1^
*T* = 298 K
0.52 × 0.23 × 0.03 mm

#### Data collection

Bruker SMART APEX CCD diffractometer
Absorption correction: multi-scan (*SADABS*; Bruker, 2000 ▶) *T*_min_ = 0.906, *T*_max_ = 0.989
9192 measured reflections
3149 independent reflections
1935 reflections with *I* > 2σ(*I*)
*R*_int_ = 0.063

#### Refinement

*R*[*F* ^2^ > 2σ(*F* ^2^)] = 0.085
*wR*(*F* ^2^) = 0.192
*S* = 1.10
3149 reflections
182 parameters
H-atom parameters constrained
Δρ_max_ = 0.37 e Å^−3^
Δρ_min_ = −0.21 e Å^−3^

### 

Data collection: *SMART* (Bruker, 2000 ▶); cell refinement: *SAINT* (Bruker, 2000 ▶); data reduction: *SAINT*; program(s) used to solve structure: *SHELXS97* (Sheldrick, 2008 ▶); program(s) used to refine structure: *SHELXL97* (Sheldrick, 2008 ▶); molecular graphics: *SHELXTL* (Sheldrick, 2008 ▶); software used to prepare material for publication: *SHELXTL*, *PARST* (Nardelli, 1995 ▶) and *PLATON* (Spek, 2009 ▶).

## Supplementary Material

Crystal structure: contains datablock(s) I, global. DOI: 10.1107/S1600536811025013/hb5920sup1.cif

Structure factors: contains datablock(s) I. DOI: 10.1107/S1600536811025013/hb5920Isup2.hkl

Supplementary material file. DOI: 10.1107/S1600536811025013/hb5920Isup3.cml

Additional supplementary materials:  crystallographic information; 3D view; checkCIF report

## Figures and Tables

**Table 1:** Hydrogen-bond geometry (Å, °)

*D*—H⋯*A*	*D*—H	H⋯*A*	*D*⋯*A*	*D*—H⋯*A*
N1—H1⋯S1^i^	0.86	2.73	3.411 (4)	137

Symmetry code: (i) 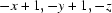.

**Fig. 1.**
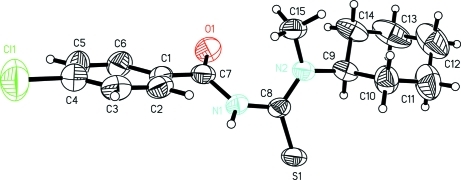
The molecular structure of the title compound with displacement ellipsoids drawn at the 50% probability level.

**Fig. 2.**
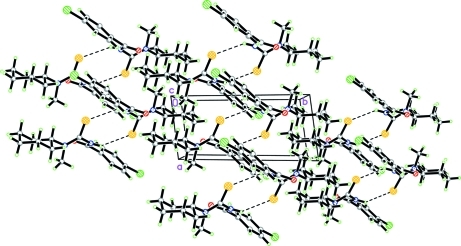
A packing diagram of the title compound down the c-axis showing the intermolecular hydrogen bonds N—H···S (-x+1, -y + 1, -z).

## supplementary crystallographic information

### Comment

The title compound, (I), is a thiourea derivative analogous to our previously reported compounds (Al-abbasi & Kassim, 2011; Nasir *et al.*, 2011). The thiono S and the carbonyl O adopt a gauche conformation at a partially double N1—C8 bond with C7—N1—C8—S1 torsion angle of -124.4 (3)°. The dihedral angle between the mean planes of the thiourea (S1/N1/N2/C8) and the amide group (O1/N1/C1/C7/C8) is 58.07 (17)°. The cyclohexane has a chair corformation and the mean planes of (C9/C10/C11/C12/C13/C14) and the 4-chlorobenzoyl (Cl1/C1/C2/C3/C4/C5/C6/C7) fragments make an angle of 26.8 (2)°.

In the crystal, intermolecular N1—H···S1 hydrogen bond links the molecules into a one dimentional polymeric structure parallel to the *a*-axis.

### Experimental

The title compound was prepared according to a previously reported compound (Hassan *et al.*, 2008).  Colourless plates of (I) were obtained by a slow evaporation of ethanolic solution at room temperature (yield 80%).

### Refinement

All H atoms were postioned geometrically with C—H bond lengths in the range 0.93 - 0.97 Å and N—H bond of 0.86 Å,.and refined in the riding model approximation with *U*_iso_(H)=1.2*U*_eq_(C,*N*), except for methyl group where *U*_iso_(H)= 1.5*U*_eq_(C).

### Crystal data

**Table:**

C_15_H_19_ClN_2_OS	*Z* = 2
*M**_r_* = 310.83	*F*(000) = 328
Triclinic, *P*1	*D*_x_ = 1.288 Mg m^−^^3^
Hall symbol: -P 1	Melting point = 418–420 K
*a* = 5.042 (2) Å	Mo *K*α radiation, λ = 0.71073 Å
*b* = 11.368 (4) Å	Cell parameters from 1114 reflections
*c* = 15.139 (6) Å	θ = 1.9–26.0°
α = 69.865 (7)°	µ = 0.37 mm^−^^1^
β = 82.698 (8)°	*T* = 298 K
γ = 80.702 (8)°	Plate, colourless
*V* = 801.7 (5) Å^3^	0.52 × 0.23 × 0.03 mm

### Data collection

**Table:**

Bruker SMART APEX CCD diffractometer	3149 independent reflections
Radiation source: fine-focus sealed tube	1935 reflections with *I* > 2σ(*I*)
graphite	*R*_int_ = 0.063
ω scan	θ_max_ = 26.0°, θ_min_ = 1.9°
Absorption correction: multi-scan (*SADABS*; Bruker, 2000)	*h* = −6→6
*T*_min_ = 0.906, *T*_max_ = 0.989	*k* = −14→14
9192 measured reflections	*l* = −18→18

### Refinement

**Table:**

Refinement on *F*^2^	Primary atom site location: structure-invariant direct methods
Least-squares matrix: full	Secondary atom site location: difference Fourier map
*R*[*F*^2^ > 2σ(*F*^2^)] = 0.085	Hydrogen site location: inferred from neighbouring sites
*wR*(*F*^2^) = 0.192	H-atom parameters constrained
*S* = 1.10	*w* = 1/[σ^2^(*F*_o_^2^) + (0.0826*P*)^2^ + 0.0972*P*] where *P* = (*F*_o_^2^ + 2*F*_c_^2^)/3
3149 reflections	(Δ/σ)_max_ < 0.001
182 parameters	Δρ_max_ = 0.37 e Å^−^^3^
0 restraints	Δρ_min_ = −0.21 e Å^−^^3^

### Special details

**Table:**

Geometry. All e.s.d.'s (except the e.s.d. in the dihedral angle between two l.s. planes) are estimated using the full covariance matrix. The cell e.s.d.'s are taken into account individually in the estimation of e.s.d.'s in distances, angles and torsion angles; correlations between e.s.d.'s in cell parameters are only used when they are defined by crystal symmetry. An approximate (isotropic) treatment of cell e.s.d.'s is used for estimating e.s.d.'s involving l.s. planes.
Refinement. Refinement of *F*^2^ against ALL reflections. The weighted *R*-factor *wR* and goodness of fit *S* are based on *F*^2^, conventional *R*-factors *R* are based on *F*, with *F* set to zero for negative *F*^2^. The threshold expression of *F*^2^ > σ(*F*^2^) is used only for calculating *R*-factors(gt) *etc*. and is not relevant to the choice of reflections for refinement. *R*-factors based on *F*^2^ are statistically about twice as large as those based on *F*, and *R*- factors based on ALL data will be even larger.

### Fractional atomic coordinates and isotropic or equivalent isotropic displacement parameters (Å^2^)

**Table:**

	*x*	*y*	*z*	*U*_iso_*/*U*_eq_	
S1	0.3915 (2)	0.34433 (9)	0.12184 (8)	0.0545 (4)	
Cl1	1.2888 (4)	0.63599 (15)	−0.45045 (10)	0.1074 (6)	
N1	0.7565 (6)	0.3373 (3)	−0.0182 (2)	0.0470 (8)	
H1	0.7996	0.4111	−0.0278	0.056*	
N2	0.7860 (6)	0.1587 (3)	0.1141 (2)	0.0436 (8)	
O1	0.7203 (6)	0.1941 (3)	−0.0894 (2)	0.0647 (9)	
C1	0.9200 (8)	0.3791 (3)	−0.1813 (3)	0.0453 (10)	
C8	0.6580 (8)	0.2720 (3)	0.0727 (3)	0.0434 (10)	
C7	0.7916 (8)	0.2943 (4)	−0.0951 (3)	0.0496 (10)	
C2	1.1049 (8)	0.4556 (4)	−0.1799 (3)	0.0511 (10)	
H2	1.1513	0.4549	−0.1221	0.061*	
C9	0.6886 (8)	0.0807 (3)	0.2086 (3)	0.0500 (10)	
H9	0.4977	0.1115	0.2179	0.060*	
C3	1.2217 (9)	0.5326 (4)	−0.2615 (3)	0.0593 (12)	
H3	1.3486	0.5823	−0.2593	0.071*	
C10	0.8322 (9)	0.0976 (4)	0.2850 (3)	0.0642 (12)	
H10A	0.8101	0.1863	0.2789	0.077*	
H10B	1.0233	0.0699	0.2772	0.077*	
C6	0.8558 (9)	0.3803 (4)	−0.2693 (3)	0.0614 (12)	
H6	0.7376	0.3271	−0.2722	0.074*	
C15	1.0483 (8)	0.1113 (4)	0.0754 (3)	0.0561 (11)	
H15A	1.0200	0.0601	0.0393	0.084*	
H15B	1.1572	0.0614	0.1262	0.084*	
H15C	1.1382	0.1813	0.0354	0.084*	
C4	1.1485 (10)	0.5351 (4)	−0.3466 (3)	0.0668 (13)	
C5	0.9673 (10)	0.4596 (5)	−0.3505 (3)	0.0718 (14)	
H5	0.9201	0.4623	−0.4086	0.086*	
C14	0.7051 (9)	−0.0591 (4)	0.2195 (4)	0.0708 (14)	
H14A	0.6045	−0.0679	0.1721	0.085*	
H14B	0.8915	−0.0930	0.2098	0.085*	
C12	0.7376 (12)	−0.1166 (6)	0.3931 (4)	0.108 (2)	
H12A	0.6573	−0.1624	0.4547	0.130*	
H12B	0.9254	−0.1518	0.3886	0.130*	
C13	0.5919 (11)	−0.1324 (5)	0.3164 (5)	0.100 (2)	
H13A	0.6101	−0.2212	0.3229	0.120*	
H13B	0.4015	−0.1031	0.3240	0.120*	
C11	0.7191 (12)	0.0218 (6)	0.3827 (4)	0.0969 (18)	
H11A	0.8191	0.0306	0.4303	0.116*	
H11B	0.5322	0.0549	0.3927	0.116*	

### Atomic displacement parameters (Å^2^)

**Table:**

	*U*^11^	*U*^22^	*U*^33^	*U*^12^	*U*^13^	*U*^23^
S1	0.0603 (7)	0.0371 (6)	0.0621 (7)	0.0054 (5)	−0.0001 (5)	−0.0182 (5)
Cl1	0.1343 (14)	0.1065 (12)	0.0655 (9)	−0.0256 (10)	0.0032 (9)	−0.0077 (8)
N1	0.065 (2)	0.0364 (17)	0.045 (2)	−0.0057 (15)	−0.0051 (17)	−0.0215 (15)
N2	0.0444 (19)	0.0371 (17)	0.051 (2)	0.0010 (14)	−0.0094 (16)	−0.0180 (15)
O1	0.090 (2)	0.0534 (18)	0.065 (2)	−0.0119 (16)	−0.0157 (17)	−0.0333 (16)
C1	0.049 (2)	0.043 (2)	0.046 (2)	0.0083 (18)	−0.0065 (19)	−0.0231 (19)
C8	0.051 (2)	0.038 (2)	0.050 (2)	−0.0032 (18)	−0.010 (2)	−0.0238 (19)
C7	0.053 (3)	0.043 (2)	0.057 (3)	0.0087 (19)	−0.016 (2)	−0.025 (2)
C2	0.051 (3)	0.054 (2)	0.055 (3)	0.001 (2)	−0.006 (2)	−0.030 (2)
C9	0.042 (2)	0.039 (2)	0.067 (3)	−0.0003 (17)	−0.009 (2)	−0.016 (2)
C3	0.057 (3)	0.056 (3)	0.070 (3)	−0.003 (2)	−0.006 (2)	−0.029 (2)
C10	0.078 (3)	0.057 (3)	0.054 (3)	−0.015 (2)	−0.007 (2)	−0.010 (2)
C6	0.074 (3)	0.066 (3)	0.055 (3)	−0.004 (2)	−0.015 (2)	−0.033 (2)
C15	0.050 (3)	0.056 (2)	0.069 (3)	0.011 (2)	−0.014 (2)	−0.033 (2)
C4	0.073 (3)	0.064 (3)	0.056 (3)	0.002 (3)	−0.003 (3)	−0.017 (2)
C5	0.085 (4)	0.087 (4)	0.047 (3)	0.002 (3)	−0.018 (3)	−0.028 (3)
C14	0.059 (3)	0.037 (2)	0.112 (4)	−0.002 (2)	−0.019 (3)	−0.016 (3)
C12	0.080 (4)	0.093 (5)	0.103 (5)	−0.011 (3)	0.005 (4)	0.024 (4)
C13	0.062 (3)	0.045 (3)	0.163 (6)	−0.013 (2)	−0.003 (4)	0.003 (3)
C11	0.106 (5)	0.104 (5)	0.061 (3)	−0.018 (4)	−0.004 (3)	0.000 (3)

### Geometric parameters (Å, °)

**Table:**

S1—C8	1.687 (4)	C10—H10A	0.9700
Cl1—C4	1.739 (5)	C10—H10B	0.9700
N1—C8	1.391 (5)	C6—C5	1.365 (6)
N1—C7	1.391 (5)	C6—H6	0.9300
N1—H1	0.8600	C15—H15A	0.9600
N2—C8	1.321 (4)	C15—H15B	0.9600
N2—C9	1.470 (5)	C15—H15C	0.9600
N2—C15	1.474 (5)	C4—C5	1.370 (6)
O1—C7	1.221 (4)	C5—H5	0.9300
C1—C2	1.381 (5)	C14—C13	1.507 (7)
C1—C6	1.406 (5)	C14—H14A	0.9700
C1—C7	1.474 (5)	C14—H14B	0.9700
C2—C3	1.370 (6)	C12—C11	1.515 (8)
C2—H2	0.9300	C12—C13	1.524 (8)
C9—C10	1.520 (6)	C12—H12A	0.9700
C9—C14	1.530 (5)	C12—H12B	0.9700
C9—H9	0.9800	C13—H13A	0.9700
C3—C4	1.375 (6)	C13—H13B	0.9700
C3—H3	0.9300	C11—H11A	0.9700
C10—C11	1.524 (6)	C11—H11B	0.9700
			
C8—N1—C7	126.1 (3)	N2—C15—H15A	109.5
C8—N1—H1	117.0	N2—C15—H15B	109.5
C7—N1—H1	117.0	H15A—C15—H15B	109.5
C8—N2—C9	120.4 (3)	N2—C15—H15C	109.5
C8—N2—C15	122.6 (3)	H15A—C15—H15C	109.5
C9—N2—C15	116.5 (3)	H15B—C15—H15C	109.5
C2—C1—C6	118.3 (4)	C5—C4—C3	120.9 (4)
C2—C1—C7	123.2 (4)	C5—C4—Cl1	119.9 (4)
C6—C1—C7	118.5 (4)	C3—C4—Cl1	119.2 (4)
N2—C8—N1	116.8 (3)	C6—C5—C4	120.3 (4)
N2—C8—S1	125.5 (3)	C6—C5—H5	119.9
N1—C8—S1	117.8 (3)	C4—C5—H5	119.9
O1—C7—N1	121.5 (4)	C13—C14—C9	110.6 (4)
O1—C7—C1	124.2 (4)	C13—C14—H14A	109.5
N1—C7—C1	114.4 (3)	C9—C14—H14A	109.5
C3—C2—C1	121.6 (4)	C13—C14—H14B	109.5
C3—C2—H2	119.2	C9—C14—H14B	109.5
C1—C2—H2	119.2	H14A—C14—H14B	108.1
N2—C9—C10	111.3 (3)	C11—C12—C13	110.4 (5)
N2—C9—C14	113.4 (4)	C11—C12—H12A	109.6
C10—C9—C14	110.9 (4)	C13—C12—H12A	109.6
N2—C9—H9	107.0	C11—C12—H12B	109.6
C10—C9—H9	107.0	C13—C12—H12B	109.6
C14—C9—H9	107.0	H12A—C12—H12B	108.1
C2—C3—C4	119.0 (4)	C14—C13—C12	111.1 (4)
C2—C3—H3	120.5	C14—C13—H13A	109.4
C4—C3—H3	120.5	C12—C13—H13A	109.4
C9—C10—C11	110.8 (4)	C14—C13—H13B	109.4
C9—C10—H10A	109.5	C12—C13—H13B	109.4
C11—C10—H10A	109.5	H13A—C13—H13B	108.0
C9—C10—H10B	109.5	C12—C11—C10	111.0 (5)
C11—C10—H10B	109.5	C12—C11—H11A	109.4
H10A—C10—H10B	108.1	C10—C11—H11A	109.4
C5—C6—C1	120.0 (4)	C12—C11—H11B	109.4
C5—C6—H6	120.0	C10—C11—H11B	109.4
C1—C6—H6	120.0	H11A—C11—H11B	108.0

### Hydrogen-bond geometry (Å, °)

**Table:**

*D*—H···*A*	*D*—H	H···*A*	*D*···*A*	*D*—H···*A*
N1—H1···S1^i^	0.86	2.73	3.411 (4)	137

Symmetry codes: (i) −*x*+1, −*y*+1, −*z*.
